# Cross-sectional associations between maternal parenting styles, physical activity and screen sedentary time in children

**DOI:** 10.1186/s12889-017-4784-8

**Published:** 2017-09-29

**Authors:** K. E. Van der Geest, S. Y. M. Mérelle, G. Rodenburg, D. Van de Mheen, C. M. Renders

**Affiliations:** 1 GGD Kennemerland, Zijlweg 200, 2015 CK Haarlem, The Netherlands; 2 IVO, Heemraadssingel 194, 3021 DM Rotterdam, The Netherlands; 3 Erasmus Medical Center Rotterdam, P.O. Box 2040, 3000 CA Rotterdam, The Netherlands; 4 Tranzo Scientific Center for care and welfare, Tilburg University, Warandelaan 2, 5037 AB Tilburg, The Netherlands; 5 Department of Health Sciences and Amsterdam Public Health research institute, Vrije Universiteit Amsterdam, the Netherlands, Van der Boechorststraat 7, 1081 BT Amsterdam, the Netherlands

**Keywords:** Physical activity, Screen sedentary time, Parenting styles, Children

## Abstract

**Background:**

Children’s activity level, including physical activity (PA) and screen sedentary time (SST), is influenced by environmental factors in which parents play a critical role. Different types of parenting styles may influence children’s activity level. Inconsistent results were found on the association between parenting styles and PA, and few studies tested the association between parenting styles and SST. This study examined the association between parenting styles, PA and SST and the modifying effect of children’s gender and maternal educational level on these associations.

**Methods:**

Cross-sectional data were collected from parents of children aged 8–11 years old who completed a web-based non-standardized questionnaire (*N* = 4047). Since 85% of the questionnaires were filled in by mothers, parenting styles are mainly reported by mothers. Multiple linear regression techniques were used to assess the associations between parenting styles (authoritative, permissive, authoritarian and neglectful), and PA and SST (mean min/day). The modifying effect of children’s gender and maternal educational level on these associations was explored. *P* values ≤.0125 were considered as statistically significant based on the Bonferroni correction for four primary analyses.

**Results:**

The neglectful parenting style was most widely used (35.3%), while the authoritarian style was least common (14.8%). No significant association was found between parenting styles and PA level. As regards SST, an authoritative parenting style was significantly associated with lower SST in boys while a neglectful parenting style was significantly associated with higher SST in both boys and girls. When the mother had a medium educational level, an authoritative parenting style was significantly associated with lower SST while neglectful parenting was significantly associated with higher SST.

**Conclusions:**

No association was found between parenting styles and PA. However, an authoritative parenting style was associated with a reduction in SST and a neglectful parenting style with an increase in SST, especially in boys and in children whose mother had a medium education level. Future studies of parenting practices are needed to gain more insight into the role of parents in children’s PA and SST levels, as a basis for the development of interventions tailored to support parents in stimulating PA and reducing SST in children.

## Background

In addition to a healthier diet, an increase in physical activity (PA) contributes to the prevention of overweight. It also has a positive effect on various aspects of mental health, such as self-concept, anxiety and depression [1, 2]. Despite the health benefits of PA, children often do not engage in 60 min physical activity per day which should be either moderate- or vigorous-intensity aerobic physical activity according to youth physical activity guidelines [3]. Not only has the time spent on PA decreased in the past decennia, but an increase in the time children spend on screen sedentary time (SST) has been reported [4–6]*.* SST is defined as time that children spend on screen-based sedentary behavior such as television watching and playing computer games [7]. PA and SST are independent behaviors; in other words, a high level of sedentary behavior is not necessarily equivalent to a lack of PA [5, 8]. Furthermore, high levels of SST may lead to an increased risk of morbidity and mortality regardless of the PA level [9, 10].

Several studies suggest that environmental factors such as the physical and social setting can have an important influence on children’s behavior [11–13]. Parents play an important role in the child’s environment and are especially important in influencing children’s behavioral patterns, such as PA and SST, in the early years of life [14, 15]. They play a critical role in developing and shaping their children’s activity patterns and preferences within the family context [15]. Various mechanisms may underpin parental impact on such childhood activities as active play and watching TV. Parenting practices such as logistic and emotional support and acting as role models are likely to be related to children’s PA and SST levels [15–17]. In addition to the way parents may influence their children’s activity behavior by parenting practices, different parenting styles can be distinguished.

Parenting style can be defined as a dispositional method of parenting toward the child that creates an emotional climate in which the parents’ behavior is expressed [18, 19]. Four prototypes of parenting styles may be distinguished by various combinations of support and behavioral control [18, 19]*.* The first style is called authoritative parenting, which combines high support and high behavioral control*.* According to Baumrind (1966), “Authoritative parents enforce their own perspective as an adult, but recognise the child's individual interests and special ways” [20]. The second style, permissive parenting, is characterized by high support and low behavioral control. Permissive parents have an affirmative, non-punitive attitude toward the child’s actions and desires. Thirdly, authoritarian parenting is characterized by low support and high behavioral control. Children are expected to follow the strict rules laid down by the parents. Authoritarian parents often fail to explain the reasons behind their instructions [19]*.* Finally, neglectful parenting is characterized by low support and low behavioral control. Parents show little responsiveness to children’s needs and wishes in this parenting style, and communication between parent and child is minimal [21]*.*

Studies on the effect of parenting styles on PA gave inconsistent results due to differences in conceptualization and in the type of physiological or self-reported measurements used [22]. These studies do however show that the effect of parenting styles on PA appears to be gender dependent. Authoritative and permissive styles were positively associated with the frequency and intensity of PA in girls, while authoritarian parenting style was positively related with PA in boys [23–25]. Furthermore, mothers with higher levels of education are more likely to engage in health-promoting behavior, such as encouraging their children’s PA [26]. Only three studies examined the relation between parenting styles and SST. They indicated that authoritarian and permissive parenting were associated with greater SST [18, 25, 27].

Due to inconclusive results the current tested the association between parenting styles and PA [24, 25]. Furthermore, only a few studies tested the association between parenting styles and SST [27, 28]. Previous studies indicated that sociodemographic characteristics such as child gender and maternal educational level are associated with both parenting styles and children’s SST level [25, 26, 29]. However, little is known about possible moderating effects of these sociodemographic characteristics on the association between parenting styles and SST [22]. To fill these shortcomings the present study aimed to examine the association between parenting styles, PA and SST in a large representative sample of young children aged between eight and eleven years. We also explored whether the association between parenting styles, PA, and SST is modified by children’s gender and maternal educational level.

## Methods

### Study design and procedure

This study was based on secondary analysis of data derived from the ‘Kindermonitor’ (‘Children’s monitor’) cross-sectional survey carried out in 2014 by the Municipal Health Services of Kennemerland, in the Midwest of the Netherlands [30]. This survey was designed to gain more insight into the health status and health-related characteristics of children aged between three and eleven living in that part of the Netherlands. While setting up this study we found that 19% of children aged three year old had high levels of SST as opposed to 44% of the children aged eight till eleven. Besides, we found that in the age category 4–7 years old 72% of the children were highly physically active as opposed to 83% in the age category 8–11 years old [31]. These relatively high levels of both PA and SST gave rise to our research aim to examine the relationship between parental styles and children’s PA and SST level in 8–11 years old children. Data collection took place between October and December 2014. Participants were randomly sampled from the municipal registry. Parents or carers (from now on referred to simply as parents) of approximately 27,000 children aged between three and eleven were invited to fill in a web-based non-standardized questionnaire. Children were excluded if they lived in institutions for the care of children with mental or other disability. In total, 10,170 of the parents invited participated (response rate 38%). A total of 104 questionnaires were excluded because the answers provided were incomplete or because the parents had moved to another municipality. This left 10,066 valid questionnaires that were used for analysis. Of these, 4047 questionnaires referred to children aged 8–11. The response rate for this group was 37%. Approval of the Medical Ethics Committee was not needed, because this study was based on a secondary analysis of anonymous survey data, collected as part of routine youth health care in The Netherlands, and is therefore not regarded as medical research [32]. Furthermore, the Dutch law ensured that the information was only used for statistical purposes and that no other institutions could require access to the data [33]. The Kindermonitor survey was registered in the administration of the Dutch Data Protection Authority (number m1576324).

### Measurements

#### Physical activity

Children’s PA level was quantified with reference to Dutch recommendations according to which healthy exercise for children aged between four and seventeen constitutes 60 min of PA daily [31]. The questionnaire that we used followed national guidelines of the National Institute for Health and Environment (RIVM) and was carefully developed based on subject-matter knowledge, but has not been validated [34]. Children’s activity levels were assessed with the aid of questions based on four different activity situations [6]. Parents were asked how many days per week their child had gone to school on foot or by bicycle, on how many days physical education lessons were given at school, on how many days the children were engaged in sport activities at a sports club, and on how many days they played outdoors outside school hours in the past week. Questions were also asked about the time spent on these various activities. Response options included ‘less than half an hour’, ‘half an hour to one hour’, ‘one to two hours’, ‘two to three hours’, and ‘more than three hours’. Based on national guidelines of the National Institute for Health and Environment (RIVM) the average duration of physical education lessons is estimated to be 60 min [34]. The number of minutes spent on these different activities were then added up to give the total number of minutes spent on physical activity each week. This dependent variable was included in the data for analysis as a continuous variable. The PA characteristics of the study population were described based on the Dutch recommendations for healthy exercise that prescribe for children aged 4–17 to be physically active for on average at least one hour per day [35, 36]. Accordingly, children were divided into ‘minimal PA level’ (<3 h/week), ‘moderate PA level’ (3–7 h/week) and ‘high PA level’ (**≥**7 h/week) [35, 36].

#### Screen sedentary time

In the questionnaire, parents reported the levels of children’s SST defined as low levels of energy expenditure in the context of watching TV and using a computer [6]. Again, the questions were based on national guidelines of the RIVM and expert opinions, but the questions have not been psychometrically tested [34]. The questions here were similar to those used to determine PA levels. Parents were asked how many days in the past week their child had engaged in SST, including watching TV/DVD and using the computer/tablet, and how many minutes per day were devoted to this form of behavior. They were instructed not to include time spent on SST at school. The response options varied from ‘less than half an hour’ to ‘more than three hours’ per day. These times (in minutes) were added up to calculate the total SST per week, which was included in the data for analysis as a continuous variable. The SST characteristics of the study population were described in terms of a dichotomous variable, in line with international recommendations that SST should be limited to less than two hours per day [13, 37]. Children were therefore categorized as ‘low SST’ (<14 h SST per week) or ‘high SST’ (**≥**14 h SST per week) [36].

#### Parenting styles

Based on earlier work of Rodenburg and Steinberg we used a 22-item questionnaire to measure general parenting styles [37, 38]. This instrument has not been validated in a Dutch sample but showed good internal consistency: Cronbach’s alpha 0.71 for the dimension support and 0.72 for the dimension behavioral control [19, 38, 39]*.* Parents were asked to indicate how they dealt with parenting by stating to what extent they agreed with 22 statements on a five-point Likert scale ranging from complete disagreement (−2) to complete agreement (+2). Two dimensions, each described in terms of seven items, were combined to give four different parenting styles (‘authoritative’, ‘permissive’, ‘authoritarian’ and ‘neglectful’). The first dimension is support or involvement (for example, I help my child with his/her homework if he/she does not understand it). The individual scores of each of the seven items involved were then summed to produce a total score, ranging from −14 (low) to +14 (high). The second dimension is behavioral control, or strict control, which refers to laying down rules for the child’s behavior and assumes that the parent has a good knowledge of how the child spends his or her time (for example, I know *exactly* what my child is doing after school). The scores for behavioral control also ranged from −14 (low) to +14 (high). The combination of these two scores indicates the type of parenting style used: high support and high control correspond to authoritative parenting, high support and low control to permissive, low support and high control to authoritarian and low support and low control to neglectful. Parenting styles were assessed by dichotomizing the sample on each dimension to give median split. The internal consistency of the two dimensions support (α = 0.73) and behavioral control (α = 0.72) were checked and approved (α > 0.7); this indicates that we could perform the analyses with two dimensions and four parenting styles, each parent being assigned to a particular parenting style. Since 85% of the mothers filled in the questionnaire, parenting styles are mainly reported by mothers.

#### Demographics

The analyses were controlled for the determinants gender (boy/girl), maternal education level and children’s ethnicity. Maternal educational level was based on self-reported answers about their highest completed level. Three educational levels were distinguished: low (completed primary school or less, completed general secondary education or lower vocational education), medium (completed higher general secondary education, pre-university education or intermediate vocational education) and high (completed higher vocational education or university education) [40]. Only the educational level of the mother was taken into account since 85% of the questionnaires were filled in by the mother. Furthermore, 4% of the questionnaires did not give details of the father’s educational level as opposed to only 0.4% of mother’s educational level. Children’s ethnicity was assessed by asking respondents to report their own country of origin (and that of their partner), and the country of origin of their child. Following guidelines laid down by Statistics Netherlands, ethnicity was divided into native Dutch, non-native/Western and non-native/non-Western [40]. Children were classified as non-native/non-Western if at least one parent was born in Africa, Latin America, Asia (excluding Indonesia and Japan) or Turkey. Children with at least one parent born outside the Netherlands, but inside Europe (excluding Turkey) or North America, Oceania or Indonesia or Japan, were classified as non-native/Western. Children were classified as native Dutch if both parents were born in the Netherlands.

### Statistical analysis

Descriptive analyses were used to calculate the mean age of children and the prevalence of different subgroups of children’s ethnicity, PA and SST level and parenting styles, separately for child gender and maternal education level and for the total study group. Chi-square tests, independent t tests and ANOVA were then used to test group differences. *P* values of ≤0.05 were considered statistically significant. In subsequent analyses, *p* values of ≤0.0125 were considered statistically significant, based on the Bonferroni correction for four primary analyses [41]. Multiple linear regression was used to assess the association between parenting styles and PA, and between parenting styles and SST. The assumptions of normality and homoscedasticity in linear regression analysis were checked and were found to be confirmed. Each parenting style was entered separately as dichotomous variable into the linear regression model to assess the association between the specific parenting style and the outcome of interest (PA or SST). Child gender, child ethnicity and maternal educational level were entered simultaneously into the model to correct for possible confounders [5, 18, 23, 25]. Associations were then estimated for the subgroups child gender (boy/girl) and maternal educational level (low/medium/high). To this end, interaction terms for the four parenting styles with gender or educational level were added separately to the adjusted model.

## Results

### Descriptive statistics

Table 1 presents demographic characteristics of the study population (*N* = 4047), together with descriptive characteristics for PA, SST and parenting styles stratified by child gender and maternal educational level. Children were, on average, 9.6 years old (SD = 1.1), the proportion of boys was 50.7%, most children were native Dutch (79.4%) and most mothers were highly educated (49.2%). The neglectful parenting style was most often used (35.3%), followed by authoritative (30.7%), permissive (19.3%) and authoritarian (14.8%). Table I also shows gender differences between characteristics for PA and SST. In general, most children were highly physically active (82.8%), while 56.2% had low SST levels (less than 14 h per week outside school). Boys had on average 805 min per week (115 min/day) of PA, significantly more than girls with 667 min per week (95 min/day). In addition, boys spent on average 861 min per week (123 min/day) on SST which was also significantly more than the mean value of 761 min per week (109 min/day) for girls. When the mother had a medium educational level, children spent 767 min per week (110 min/day) on PA, significantly more compared to when the mother had a low educational level (106 min/day) or a high educational level (102 min/day). In addition, children’s SST level was significantly higher when their mothers had a low educational level (134 min/day) compared to children who had medium (121 min/day) or high educated mothers (106 min/day). All group differences were highly significant (*p* < 0.001).

**Table 1:** Descriptives of socio-demograhic variables, child PA, child SST and parenting styles stratified by child gender and maternal educational level

Variable	Child gender			Total		Maternal education level			Total
	Boys (N=2053)	Girls (N=1994)		(N=4047)	Low (N=598)	(N=4015)	High (N=1974)		(N=4015)
	Mean (SD)	Mean (SD)		Mean (SD)	Mean (SD)	Mean (SD)	Mean (SD)		Mean(SD)
Age [years]	9.6 (1.1)	9.6 (1.1)		9.6 (1.1)	9.7 (1.2)	9.6 (1.1)	9.6 (1.1)		9.6 (1.1)
Child ethnicity [%]	N=2052 (100)	N=1994(100)		N=4046 (100)	N=598 (100)	N=1442 (100)	N=1974 (100)	*	N=4014 (100)
Native Dutch	1643 (80.0)	1584 (79.4)		3227 (79.8)	386 (64.5)	1216 (84.3)	1619 (82.0)		3221 (80.2)
Non-native/Western	166 (8.1)	164 (8.2)		330 (8.2)	41 (6.9)	92 (6.4)	191 (9.7)		324 (8.1)
Non-native/non-Western	243 (11.8)	246 (12.3)		489 (12.1)	171 (28.6)	134 (9.3)	164 (8.3)		469 (11.7)
Child PA [%]	N=2034 (100)	N=1975 (100)	*	N=4009 (100)	N=591 (100)	N=1430 (100)	N=1960 (100)	*	N=3981 (100)
Minimal (<3 h/week)	39 (1.9)	55 (2.8)		94 (2.3)	30 (5.1)	27 (1.9)	33 (1.7)		90 (2.3)
Moderate (3-7 h/week)	220 (10.8)	374 (18.9)		594 (14.8)	111 (18.8)	207 (14.5)	270 (13.8)		588 (14.8)
High (≥7 h/week)	1775 (87.3)	1546 (78.3)		3321 (82.8)	450 (76.1)	1196 (83.6)	1657 (84.5)		3303 (83.0)
Child SST [%]	N=2026 (100)	N=1970 (100)	*	N=3996 (100)	N=590 (100)	N=1424 (100)	N=1954 (100)	*	N=3968 (100)
Low (<14 h/week)	1023 (50.5)	1221 (62.0)		2244 (56.2)	278 (47.1)	742 (52.1)	1212 (62.0)		2232 (56.3)
High (≥14 h/week)	1003 (49.5)	749 (38.0)		1752 (43.8)	312 (52.9)	682 (47.9)	742 (38.0)		1736 (43.8)
Parenting style [%]	N=1874 (100)	N=1815 (100)		N=3689 (100)	N=523 (100)	N=1310 (100)	N=1829 (100)		N=3662 (100)
Authoritative	575 (30.7)	557 (30.7)		1132 (30.7)	149 (28.5)	408 (31.1)	569 (31.1)		1126 (30.7)
Permissive	377 (20.1)	334 (18.4)		711 (19.3)	97 (18.5)	276 (21.1)	330 (18.0)		703 (19.2)
Authoritarian	263 (14.0)	282 (15.5)		545 (14.8)	77 (14.7)	181 (13.8)	285 (15.6)		543 (14.8)
Neglectful	659 (35.2)	642 (35.4)		1301 (35.3)	200 (38.2)	445 (34.0)	645 (35.3)		1290 (35.2)

Note: (SD) = standardized deviation

Note: * Chi-square tests, independent t tests and ANOVA, *p* ≤ .05

Note: Due to rounding the sum of the percentage in each category can be somewhat higher or lower than 100%, for example 99.9% or 100.1%

### Association between parenting style and physical activity

In Table 2 neither the unadjusted nor the adjusted model showed any statistically significant association between parenting styles and PA level, and no significant interaction terms were found between parenting styles and child gender or maternal educational level. Secondary analyses, in which the association of the two dimensions support (B^2^ = 13.71, Std. Error = 11.48, *p* = 0.23) and behavioral control (B^2^ = −6.58, Std. Error = 11.64, *p* = 0.57) with PA was tested for each dimension separately, did not change these outcomes.

**Table 2:** Results of linear regression analyses of parenting style on PA and SST level in unadjusted and adjusted model

Parenting style^a^	PA level model 1^b^		PA level model 1^c^		SST level model 2^b^		SST level model 2^c^	
	B^2^	Std. Error	B^2^	Std. Error	B^2^	Std. Error	B^2^	Std. Error
Authoritative	0.61	12.69	1.56	12.45	−52.84*	15.52	−48.80*	15.20
Permissive	17.20	14.81	7.93	14.62	13.98	18.14	5.52	17.86
Authoritarian	−16.91	16.47	−14.77	16.20	−20.99	20.19	−10.75	19.82
Neglectful	−2.97	12.24	1.32	12.04	51.26*	14.98	47.84*	14.70

Note: B^2^ = unstandardized regression coefficient; Std. Error = Standard error; *p ≤ .0125

^a^Association between four different parenting styles, e.g., authoritative vs. non-authoritative. ^b^Unadjusted PA/SST model. ^c^Model adjusted for child gender, child ethnicity and maternal educational level

### Association between parenting style and screen sedentary time

In Table 3 several parenting styles revealed significant effects related to SST levels in children. The authoritative and neglectful parenting styles were associated with decreased and increased SST, respectively, in both the unadjusted and the adjusted model. Gender stratification revealed statistically significant results for boys and girls. The neglectful parenting style was significantly associated with a higher SST level in boys (B^2^ = 52.76, Std. Error = 16.68) and girls (B^2^ = 46.06, Std. Error = 16.59), while the authoritative style was significantly associated with a lower SST level in boys only (B^2^ = −54.26, Std. Error = 17.86). Stratification for maternal educational level showed that significant associations between parenting style and SST were only found when the mother had a medium educational level. In this case, the authoritative parenting style was negatively associated with SST (B^2^ = −86.12, Std. Error = 25.31), while the neglectful style was positively associated with SST (B^2^ = 93.23, Std. Error = 24.82).

**Table 3:** Results of adjusted linear regression analyses of parenting style on SST level, stratified by child gender and maternal educational level

Parenting style^a^	SST level model 3									
	Gender^b^				Maternal educational level^c^					
	Boys		Girls		Low		Medium		High	
	B^2^	Std. Error	B^2^	Std. Error	B^2^	Std. Error	B^2^	Std. Error	B^2^	Std. Error
Authoritative	−54.26*	17.86	−34.51	17.99	−26.61	41.09	−86.12*	25.31	−28.08	21.43
Permissive	29.01	21.86	1.84	21.87	−15.39	47.77	−1.44	28.82	17.26	25.89
Authoritarian	5.42	23.14	−3.75	22.80	−1.18	52.45	−17.59	34.08	−8.94	27.51
Neglectful	52.76*	16.68	46.06*	16.59	33.33	38.17	93.28*	24.82	20.30	20.76

Note: B^2^ = unstandardized regression coefficient; Std. Error = Standard error; *p ≤ .0125

^a^Association between four different parenting styles, e.g., authoritative vs. non-authoritative, and SST level. Adjusted models for: gender, ethnicity, educational level. ^b^Model stratified by child gender. ^c^Model stratified by maternal educational level

## Discussion

### Key findings

This study examined whether parenting styles are related to children’s PA and SST behavior. Parenting styles were not associated with PA. However authoritative parenting was associated with spending less time on SST in boys and neglectful parenting was associated with spending more time on SST in both boys and girls. The maternal educational level also influenced the relation between parenting styles and SST. Authoritative parenting was associated with less time on SST in children of mothers with a medium educational level, while neglectful parenting was associated with more time on SST in such children. These relationships were not found in children of mothers with low or high educational levels.

In the current study, the majority of the children showed high levels of physical activity (≥7 h/week), with boys being significantly more active than girls. This difference is also found in other studies, and is probably amplified in older children since girls are more likely to decrease their PA level during teenage years than boys [42, 43]. Our results did not show a significant relation between parenting styles and PA. These results confirm previous research by Sleddens et al. and Vollmer et al. who stated that a certain parenting style is not an important predictor of children’s PA level and that other determinants may be more important in explaining children’s PA level [22, 44]. According to recent reviews, parenting styles may operate at a broader, more distal level whereas parenting practices may be more directly linked to child health outcomes [23]. Trost & Loprinzi tested the influence of parenting practices and parenting styles on children’s PA level [16]. Although no relation was found between parenting styles and children’s PA levels, they found that parenting practices (in particular parental support and modelling) were positively related to child activity. Other studies also clearly demonstrated that parenting practices such as modelling and support are important in encouraging PA level in children [23, 24]. For example, facilitating child involvement in sports, in particular providing logistic support to allow the child to go to places where it can engage in appropriate physical activity, can help to raise PA levels [23, 24]. In a recent study experts have defined practices that influence children’s participation in PA and have translated them in constructs that should be included in measures of physical activity parenting practices [45].

The results of the present study show that boys are more likely to engage in high levels of SST (≥14 h/week) than girls. The time that boys spent on online games might explain these results, as Mérelle et al. found in older ages that boys were at higher risk of problematic video-gaming than girls and that sedentary behavior was strongly associated with problematic video-gaming [46].

Our study showed that authoritative parenting was associated with lower SST levels in children. In boys this association was significant and in girls the association was borderline significant (*p* = 0.055). This confirms the results of other studies, indicating that the authoritative style in which parents are involved and understanding but also demanding may be an effective way of limiting SST in boys [43]. We further found that both boys and girls from families where the parents were neglectful, displaying little involvement with and warmth towards their children, spend more time on SST than children whose parents are not neglectful. This implies that parents should reach agreement with their children about permissible levels of screen time and monitor compliance with these rules in order to achieve substantial reductions in SST in their children.

Besides, differences in SST level were found for maternal education level. Descriptive results show that children’s SST level was higher when their mothers had a low educational level, and that neglectful parenting style was most frequently used in low educated mothers. Significant associations between parenting style and SST were only found when mothers had medium educational levels, however. In this subgroup, neglectful parenting was associated with higher SST levels and authoritative parenting with low SST levels. Tandon et al. showed that children from lower income households did not differ in total sedentary time from children from high income households, but they had greater access to screen activities and their daily screen time was somewhat higher compared to children of high incomes [47]. These results indicate that socioeconomic status seems important for children’s SST but other determinants than parenting styles may be more directly linked to children’s SST from mothers with low or high educational levels. It would however be premature to use these findings as a basis for the development of targeted intervention aimed at reducing children’s SST levels; further research on the role of parenting practices and styles is needed before this step can be taken.

Overall, it is interesting that parenting style was associated with SST and not with PA. This could be due to a broader knowledge and acceptance about the positive health advantages of PA among all parents independent of their parenting style while SST is a new ‘risky’ lifestyle which is more unfamiliar for parents. In addition, less is known about the negative health consequences of SST among children, and therefore, parents may have less knowledge, skills and may sense less urgency to educate their children in healthy SST behaviour and to limit screentime [48]. As a consequence, parents may adapt different styles in monitoring and restricting children’s SST. Moreover, since 85% of the questionnaires were filled in by mothers our results mainly concern maternal parenting styles. Lloyd et al. found that maternal parenting was significantly associated with screen time, while father’s parenting was specifically related to children’s physical activity. This difference in parenting between mothers and fathers might also explain the results that only associations with SST were found [49].

### Strengths and limitations

The present study has a number of strengths. It is one of the first to examine the relationship between parenting styles in general and children’s PA and SST levels in a large-scale, representative sample of children. One of the main advantages of this approach is that it allows us to examine associations involving both PA and SST levels in the same study population and to explore the influence of children’s gender and maternal educational level on these associations. Our findings also extend existing knowledge on the influence of different parenting styles: most previous studies focused on authoritative and authoritarian parenting, and did not take neglectful parenting into account. To the best of our knowledge, our study is the first to demonstrate the adverse relationship of neglectful parenting and children’s SST level. Our study also has several limitations, however. By its very nature, this secondary cross-sectional study precludes the drawing of any temporal or causal conclusions. In addition, the multiple analyses performed mean that type I errors (false positives) cannot be excluded. The likelihood of such errors is however reduced by the fact that we applied Bonferroni correction and took a conservative approach to test the associations between parenting styles and PA/SST. The fact that we based children’s PA and SST levels on parental reports might also have biased the results towards more socially desirable outcomes, leading to possible overreporting of PA levels and underreporting of SST levels [50]. In addition, we could not check for children’s activity level during physical education lessons or other physical activities. Children’s activity level in physical education lessons are often far less, than the 100% time that was used in the calculations [51]. Therefore the extent to which children participate in physical education lessons could have been overestimated [51, 52]. Nevertheless, when children get opportunities to exercise, for example in school setting, their PA level is generally higher compared to an environment in which these opportunities are not offered [52]. Furthermore, the questionnaire that we used to measure parenting styles was based on earlier research, but the instrument has not been validated in a Dutch sample. Another point of discussion is that the response rate of the survey is quite low (37%). Non-respondents may have differed in important aspects from the study population. We found in our study sample that mothers with high educational levels were overrepresented. This may affect the generalizability of our results. The distribution of non-Native/Dutch was representative of the general Dutch population [40]. Besides, parents may be ignorant of their children’s PA and SST level since they have no constant overview of their children’s behavior. Moreover, parents (mothers) with neglectful parenting may be even more prone to this type of information bias as they are likely to be less involved in their children’s activities. Furthermore, another point of discussion follows the fact that 85% of the questionnaires were filled in by the mothers. Selection bias could have occurred since highly educated mothers were overrepresented (49%) in our sample, as only 40% of the Dutch population as a whole has completed higher education. However, since no associations involving these mothers were found in our study, it is unlikely that this overrepresentation affected the results [40].

### Implications

Future research could focus on the independent and interactive effects of parenting practices and parenting styles to explain children’s PA and SST levels. Authors studying children’s dietary behavior defined parenting practices such as monitoring or restriction as ‘specific techniques or behaviors usually used to facilitate or limit ingestion of foods’ [50], but of course this definition also applies, *mutatis mutandis*, to children’s physical activity. However, research addressing the role of parenting practices in stimulating PA and reducing SST is still in its infancy. It may therefore be important for future studies to include parenting practices as well as parenting styles as determinants of PA and SST levels in children [24]. Darling & Steinberg (1993) suggested that parenting styles may moderate the association between specific parenting practices and child health outcomes [37, 45, 53] According to their hypotheses, parental practices and styles might either be additively adaptive or maladaptive or might serve to offset one another. Understanding how these two parenting concepts interact may lead to the design of more efficacious intervention strategies aimed at raising children’s PA level and lowering their SST level.

The results of the present study provide evidence of associations between parenting styles and their children’s SST level but do not confirm earlier associations that were found between parenting styles and children’s PA. Improving parenting skills may influence a child’s SST level in a favourable way. Family-based interventions, which include promoting PA and reducing SST, in which both parents and children are involved, may be the most effective way of promoting healthy active lifestyles in children [49]. Parents need to be aware of the specific aspects of their behavior that can have unintended effects on their child’s PA and SST level. Moreover, future research in this field should adopt a broader contextual approach by taking parenting practices into account in order to understand and counteract the processes leading to the development of overweight in children.

## Conclusion

This study extends our knowledge of the influence of parenting styles on PA and SST. No association was found between parenting styles and PA. However, an authoritative parenting style was associated with a reduction in SST and a neglectful parenting style with an increase in SST, especially in boys and in children whose mother had a medium education level. The association between neglectful parenting and high SST was also found in girls. Since the comparison of SST with parenting styles has been studied relatively little in comparison to other lifestyle behaviors, it is crucial to gain a better understanding of the mechanisms underlying this relationship. It would be useful in this context to learn more about the influence of parenting practices in a wider sense as well as parenting styles. Such research could provide a basis for the development of more targeted interventions aimed at supporting parents in their efforts to reduce SST and stimulate PA in their children.

## Abbreviations

PA: physical activity
SST: screen sedentary time
